# Mechanisms Driving Ground‐Dwelling Animal Diversity Responses to Urbanization Gradients: A Case Study From the Chuhe River Riparian Zone in Nanjing, China

**DOI:** 10.1002/ece3.73631

**Published:** 2026-05-05

**Authors:** Wenjing Chen, Qi Zhu, Yunfeng Yang

**Affiliations:** ^1^ College of Landscape Architecture Nanjing Forestry University Nanjing China

**Keywords:** biodiversity responses, community assembly, functional traits, urbanization

## Abstract

Urbanization is a major driver of global ecosystem change and biodiversity loss. Ground‐dwelling animals, due to their reliance on near‐ground resources and sensitivity to human disturbance, serve as important indicator taxa in urban ecosystems. Understanding the responses of ground‐dwelling animals to urbanization is therefore critical for the design and management of urban green spaces. In this study, we evaluated the responses of ground‐dwelling animal communities to urbanization along the Chuhe River riparian zone in Nanjing, China. Using camera trap data and a set of urbanization‐related variables, we examined community patterns across taxonomic, functional, and phylogenetic dimensions of diversity. The results showed that: (1) no significant differences in α diversity were detected among urban, suburban, and rural sites; (2) generalized linear models indicated that different urbanization‐related factors exerted significant and taxon‐specific effects on diversity metrics, with building height, building density, road density, and distance to the government site being key variables; and (3) RLQ analysis revealed significant associations between urbanization and functional traits, with more urbanized areas favoring species characterized by larger body size, carnivorous diets, ground‐dwelling habits, and solitary behavior. Overall, urbanization did not lead to significant changes in local diversity across categorical urbanization gradients, but influenced ground‐dwelling animal communities through specific urbanization‐related factors and trait‐based filtering processes. These findings highlight the importance of integrating multiple diversity dimensions and trait‐based perspectives into urban ecological research and planning.

## Graphical abstract

Systematic assessment of ground‐dwelling animal diversity responses along urbanization gradients in the Chuhe River riparian zone, Nanjing, China. Integration of taxonomic, functional, and phylogenetic diversity to comprehensively reveal community changes across different urban gradients. Identification of key urban drivers shaping animal communities: building height, building density, road density, and distance to government sites. Evidence that urbanization filters species traits, favoring large‐bodied, carnivorous, ground‐dwelling, and solitary species.

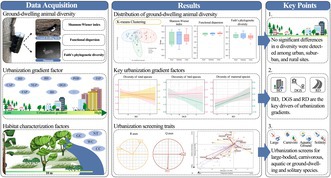

## 1 Introduction

Urbanization has emerged as a dominant driver of global ecosystem change. According to the United Nations Human Settlements Programme (UN‐Habitat 2022), nearly 70% of the world's population is expected to reside in urban areas by 2050. Rapid urban expansion often leads to habitat fragmentation and a reduction in natural habitat area, resulting in simplified and homogenized urban ecosystems and the loss of biodiversity (Aronson et al. 2017). As a fundamental component of ecosystem stability and functioning, biodiversity is vital for human well‐being, ecological security, and sustainable development. However, under the dual pressures of urbanization and climate change, global biodiversity is declining at an unprecedented rate (IPBES 2019).

Within urban ecosystems, animals that are primarily active at ground level and detectable using ground‐based camera traps—including ground‐active birds and terrestrial mammals—are particularly sensitive to habitat loss and human disturbance due to their reliance on ground‐level resources and microhabitats (Brocardo et al. 2023). Compared to arboreal or aquatic species, ground‐dwelling animals are generally more likely to encounter human activities at the surface, whereas arboreal and aquatic species are primarily affected indirectly through habitat alteration (Hursh et al. 2023). They also tend to have limited mobility and narrower ecological niches, making them especially vulnerable to urban impacts (Neate‐Clegg et al. 2023). With the rapid advancement of camera trap technology, which enables non‐invasive and all‐weather monitoring, ecological studies of ground‐dwelling species in urban settings have become increasingly feasible (Zhang et al. 2024). Camera traps, once mainly used in nature reserves, are now widely applied in urban green spaces and ecological corridors (Rega‐Brodsky and MacGregor‐Fors 2023; Blount et al. 2021). This shift offers new opportunities to assess how urbanization influences the community composition and functional structure of ground‐active bird and mammal communities.

Urbanization gradients—typically characterized by a spatial continuum from urban cores to suburban and rural outskirts—are widely used to describe variation in biodiversity and environmental conditions across urban landscapes (McKinney 2002). Numerous studies have reported that biodiversity responses to urbanization vary widely across taxa, spatial scales, and ecological contexts, with declines, weak responses, or even localized increases in diversity all being documented (Piano et al. 2020; Hastedt and Tietze 2023; Vaz et al. 2023). However, some research has shown that moderate or low levels of urbanization can enhance species richness, possibly due to increased habitat heterogeneity, remnant green spaces, or species adaptation to urban environments (Amaya‐Espinel et al. 2019; Pfeiffer et al. 2023).

Responses to urbanization vary across taxonomic groups. Therefore, interpretations of urban biodiversity patterns should be constrained within the focal taxonomic groups under investigation (McKinney 2002; Aronson et al. 2014). For instance, large carnivorous mammals have been locally extirpated in many urban areas due to severe habitat fragmentation and intense human activity (Crooks 2002; Riley et al. 2003). In contrast, adaptable omnivorous mammals such as wild boars ( *Sus scrofa* ) and raccoon dogs ( *Nyctereutes procyonoides* ) can persist in remnant urban woodlands (Tee et al. 2018; Wang et al. 2023). Among ground‐dwelling birds, species occurring in human‐modified landscapes often exhibit traits associated with urban tolerance, such as high dispersal ability, broad diets, and wide ecological niches, while body size responses may vary along urban–suburban–rural gradients (Neate‐Clegg et al. 2023). These patterns reflect strong ecological filtering in urban environments, which selectively favor species with advantageous traits, leading to reduced functional diversity and increased taxonomic homogenization (McKinney 2006; Trentanovi et al. 2013; Merckx and Van Dyck 2019). At the same time, phylogenetic diversity—which captures the evolutionary history and relatedness among species—provides complementary insights to functional diversity. Phylogenetic diversity can reveal patterns of evolutionary redundancy or uniqueness in communities and may help identify lineages that are particularly sensitive or resilient to urbanization. In urban ecosystems, phylogenetic diversity can indicate whether species losses are random or phylogenetically clustered, which has implications for ecosystem functioning, resilience, and conservation prioritization (Flynn et al. 2011).

The mechanisms by which urbanization influences animal diversity are complex, involving both overall urbanization gradients and specific environmental factors. For instance, impervious surface coverage, a key proxy for urban intensity, is closely associated with ecological issues such as the urban heat island effect, noise pollution, and habitat fragmentation (Breitbart et al. 2023). Building density and height affect nesting opportunities, resource accessibility, and predation risk, while urban green spaces provide key foraging and refuge habitats, functioning as vital ecological infrastructure for biodiversity (Reynolds et al. 2019). Other human disturbance indicators, such as road density, nighttime light intensity, and population density, also significantly shape community structure and functional composition (Møller et al. 2014; Seress et al. 2018; Chen et al. 2023).

Identifying the key drivers of biodiversity under urbanization has become a central focus in urban ecology. Socioeconomic variables like building density and population density often show negative associations with biodiversity, whereas vegetation‐related factors such as forest cover and green space ratio typically have positive effects (Callaghan et al. 2018; Hughes et al. 2022; Lepczyk et al. 2017; Rega‐Brodsky et al. 2022). However, due to the multidimensionality and spatial heterogeneity of urban environments, there is still a lack of systematic analyses examining the relative importance and mechanisms of different urbanization variables in shaping community diversity.

Moreover, urban community assembly is jointly influenced by habitat fragmentation and resource reconfiguration. Urban environmental variables can act as ecological filters, selecting for species with specific trait combinations. This filtering process may constrain functional trait diversity and, under certain conditions, promote trait homogenization, resulting in simplified community structure and reduced functional differentiation. Traits such as small body size, omnivory, high dispersal ability, and short reproductive cycles are frequently observed in highly urbanized areas (Merckx and Van Dyck 2019; Neate‐Clegg et al. 2023). Despite increasing theoretical and empirical attention to functional traits, systematic assessments of trait responses along urbanization gradients remain rare—particularly for ground‐dwelling species. Quantitative evidence on trait–environment matching and trait‐based filtering mechanisms is still limited (Hahs et al. 2023).

In summary, the responses of ground‐active bird and mammal communities to urbanization—across taxonomic, functional, and phylogenetic dimensions—remain insufficiently understood. The key environmental drivers and mechanisms underlying these responses need further elucidation. This study focuses on the Chuhe River riparian zone in Nanjing, China, a region that spans rural, suburban, and urban zones from west to east, forming a typical urbanization gradient while retaining a mosaic of natural and semi‐natural habitats. In recent years, ecological restoration initiatives have been implemented in this area, and it has been recognized as a region of ecological importance in urban planning. Using camera trap data and a suite of urbanization variables, we analyze how ground‐dwelling animal communities respond to urbanization across multiple biodiversity dimensions. Specifically, we address the following questions:

1. How do taxonomic, functional, and phylogenetic diversity of ground‐dwelling animal communities vary across urban, suburban, and rural areas?
2. Which urbanization variables are the primary drivers in community diversity?
3. Do ground‐dwelling animals exhibit directional trait filtering and structural community responses along the urbanization gradient?

## 2 Materials and Methods

### 2.1 Study Area

Nanjing (31°14′–32°37′ N, 118°22′–119°14′ E), located in southwestern Jiangsu Province in eastern China along the middle‐lower Yangtze River, has a subtropical monsoon climate with four distinct seasons, warm and humid conditions, and an annual mean temperature of 16.5°C. Average annual precipitation is 1106.5 mm, mainly from May to September, and relative humidity averages 76%. Native vegetation is dominated by deciduous broadleaf forests, alongside evergreen and coniferous species.

The Chuhe River riparian zone, a key ecological corridor in the lower Yangtze River region, extends 36.7 km west to east across a typical urban–suburban–rural gradient, encompassing diverse habitats. This area offers an ideal setting to examine how urbanization affects animal community structure. As no official boundary exists, we delineated the riparian zone based on field surveys and remote sensing, defining vegetated land within 1500 m on both sides of the river as the study area (Wu et al. 2025).

### 2.2 Ground‐Dwelling Animal Survey

**FIGURE 1:**
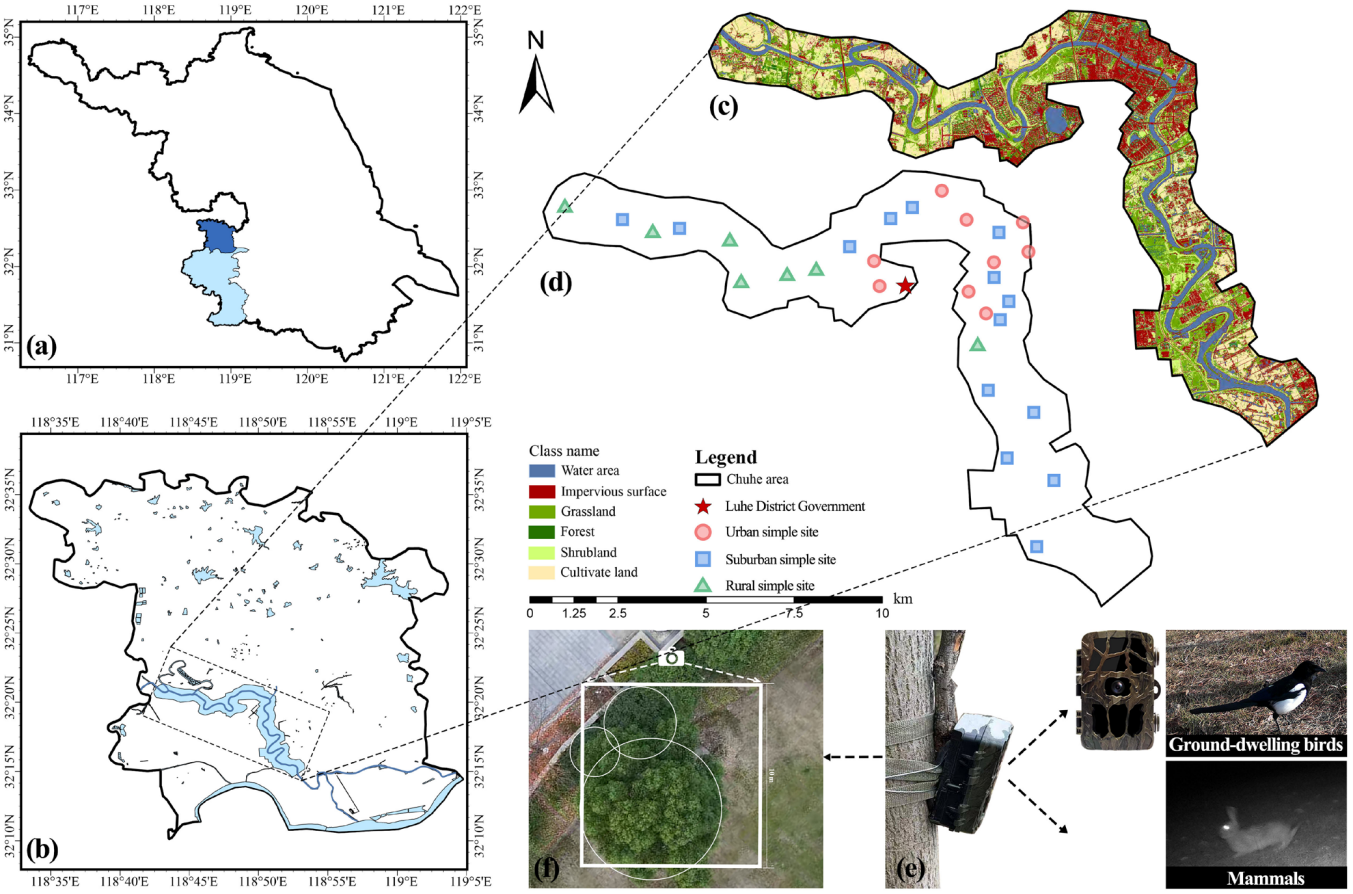
Study area and field survey design. (a) Geographic location of Luhe District, Nanjing, Jiangsu Province. (b) Location of the Chuhe River riparian zone within Luhe District. (c) Land use classification of the study area. (d) Distribution of sampling sites along the Chuhe River. (e) Placement of camera traps for ground‐dwelling animal monitoring. (f) Layout of the 10 m × 10 m vegetation survey plot for assessing local habitat variables.

A winter survey of ground‐dwelling animal diversity was conducted in the Chuhe River riparian zone of Luhe District, Nanjing, from Dec 2024 to Feb 2025. To systematically assess the effects of urbanization on ground‐dwelling animals and their functional traits, we established 30 fixed sampling sites along the Chuhe River riparian zone. These sites were evenly distributed across three urbanization categories—urban, suburban, and rural zones (Figure 1). The number of sampling sites was determined by balancing spatial representativeness and field feasibility. Previous studies have indicated that approximately 25–35 sampling sites are generally sufficient to detect reliable trends in species richness and community composition (Kays et al. 2020), suggesting that our sampling effort was adequate. Site selection prioritized relatively flat, quiet areas with minimal human disturbance. The minimum distance between any two sampling sites was 200 m to ensure spatial independence (Pyšková et al. 2018).

At each site, one camera trap (model: Forsafe H805) was mounted 30–50 cm above ground on a tree trunk, angled downward to cover an area of about 10 m radius. Cameras operated in “photo + video” mode, taking three photos and a 5 s video per trigger, with a 30 s interval to avoid redundant captures. From each image, we identified species, counted individuals, and recorded detection time. Consecutive records of the same species were considered independent if separated by more than 10 min (Tanwar et al. 2021). To standardize sampling effort, each camera was deployed continuously for 4 days (96 h) per site and operated 24 h per day. Due to limitations in camera availability and field personnel, only six cameras could be deployed simultaneously on any given survey day. Cameras were therefore rotated among the 30 sampling sites over multiple survey rounds throughout the study period. In total, this sampling design resulted in an overall camera‐trapping effort of 120 camera‐days.

Species abundance at each site was estimated using the number of independent detections per species. Independent detections were first filtered to reduce repeated records of the same individual and then aggregated at the site level to represent relative local abundance. This approach provides a standardized proxy for abundance while minimizing pseudoreplication in camera trap data.

### 2.3 Urbanization Variables

Based on a review of urban ecological studies and the urbanization characteristics of Luhe District, Nanjing, we selected two categories of urbanization indicators: human disturbance and land cover. All metrics were quantified within a 200 m buffer around each sampling site.

For human disturbance, six representative variables were selected: building density (BD), mean building height (BH), road density (RD), intensity of nighttime light pollution (NLP), population density (POD), and distance to the government center (DGS). BD, BH, and RD were derived from the OpenStreetMap database (2020; https://www.openstreetmap.org/). NLT was obtained from the 2020 VIIRS annual composite (Earth Observation Group 2020), and POD from the 2020 WorldPop dataset (2020; 1 km resolution; https://www.worldpop.org/). DGS was calculated as the Euclidean distance from each sampling site to the Luhe District Government Office (32°19′33″ N, 118°49′02″ E) using Google Earth (Google 2025).

Land cover data were extracted from 0.5 m Jilin‐1 satellite imagery acquired on December 31, 2024. After orthorectification, radiometric correction, fusion, and mosaicking, land cover was classified into six types: forest (FAP), shrubland (SAP), grassland (GAP), cultivated land (CAP), impervious surface (IAP), and water (WAP), following the National Standard of China for land use classification (GB/T 21010–2017). Classification accuracy, assessed with 100 random points, yielded a Kappa coefficient of 0.836, indicating satisfactory accuracy. All spatial analyses were performed in ArcGIS Pro 3.16 (Esri 2024).

### 2.4 Local Habitat Variables

During the camera trapping period, we simultaneously conducted vegetation surveys within a 10 m × 10 m plot located in front of each sampling site (Zhang et al. 2024). The following local habitat characteristics were recorded: canopy cover (CC), grassland cover (GC), water cover (WC), and the number of trees (NT). The choice of a 10 × 10 m plot was primarily constrained by the narrow and heterogeneous riparian habitats of the Chuhe River Riparian zone, where larger plots (e.g., 20 × 20 m) would likely span multiple habitat types and thus reduce sampling precision. In addition, this scale closely corresponds to the effective detection range of the infrared cameras, as most animal activities were captured within approximately 10 m of the camera view (Randler and Kalb 2018). These microhabitat variables were subsequently integrated with the remotely sensed urbanization indicators to support joint analytical models linking species occurrence, environmental conditions, and functional traits.

### 2.5 Diversity Metrics and Response Variables

We identified all ground‐dwelling animals captured by the camera traps and recorded the number of individuals and their frequency of occurrence for each species. Individuals clearly associated with human ownership, such as those wearing collars, being accompanied by humans, or showing restricted activity around residential areas, were excluded from all analyses. Free‐ranging cats and dogs that were independently detected by camera traps and exhibited no signs of ownership were retained in the dataset. These individuals were treated as free‐ranging carnivores rather than pets, as they move freely across urban landscapes and interact ecologically with other wildlife.

We compiled four functional traits for each species: diet, nesting location, social behavior, and body size (Myhrvold et al. 2015; Wilman et al. 2014). These traits were selected because they represent key ecological dimensions related to resource use, mobility, and behavioral strategies that determine species' responses to urbanization. Specifically, species were classified into three dietary categories (herbivores, carnivores, and omnivores), three vertical habitat types (arboreal, terrestrial, and aquatic), two social categories (social vs. solitary), and three body size classes (small, medium, and large).

To assess phylogenetic diversity, we obtained mitochondrial protein‐coding gene sequences of cytochrome c oxidase subunit III (COX3) for all target species from the National Center for Biotechnology Information (NCBI, n.d.; https://www.ncbi.nlm.nih.gov/). COX3 was selected because it is widely available across both bird and mammal taxa and provides sufficient phylogenetic signal for comparative analyses (Sayers et al. 2022). Amino acid sequences were aligned using the MUSCLE algorithm implemented in MEGA11 software (Tamura et al. 2021). A phylogenetic tree was constructed using the Maximum Likelihood method with 1000 bootstrap replicates and exported in Newick format for subsequent analyses. The phylogenetic tree was visualized and formatted using the chiplot package (Xie et al. 2023). The phylogenetic tree used in the analysis is provided in Data S1.

Based on the species occurrence, functional trait matrix, and phylogenetic tree constructed above, we quantified multiple dimensions of community diversity at each sampling site. We calculated Shannon–Wiener index (Shannon), functional dispersion (FDis), and phylogenetic diversity (PD) for the ground‐dwelling animal community at each sampling site. Shannon diversity was computed using the vegan R package (Oksanen et al. 2012) to represent species richness and evenness. Functional diversity was measured by FDis, defined as the mean distance of species to the centroid in trait space (Laliberté and Legendre 2010). FDis was selected because it is independent of species richness and less sensitive to unequal abundances, making it suitable for communities with varying species numbers. Phylogenetic diversity was quantified using Faith's PD metric, with tree processing conducted using the rtree R package (Li 2023), thereby incorporating evolutionary relationships into community‐level diversity assessment.

### 2.6 Statistical Analyses

Each camera deployment was treated as an independent sampling unit, resulting in a total of 30 valid sites used for analyzing animal community structure and functional traits. All diversity indices were log‐transformed prior to analysis [log(*x* + 1)] to reduce right‐skewness and improve distributional properties for Gaussian modeling. Environmental variables were standardized using z‐score transformation to remove unit effects.

To examine the effects of urbanization on ground‐dwelling animal communities, we first conducted Pearson correlation analyses to identify and exclude highly collinear variables (|*r*| > 0.70) and those weakly associated with diversity (e.g., WAP and GAP), in order to minimize redundancy and reduce the risk of multicollinearity (Dormann et al. 2013). We then conducted a principal component analysis (PCA) based on 10 urban‐related variables (BD, BH, RD, POD, NLP, DGS, FAP, SAP, CAP, and IAP), after excluding WAP and GAP due to their weak associations with diversity indices. The first two principal components (PC1 and PC2), which explained 42.59% and 24.96% of the total variance, respectively, were extracted as composite axes representing the urbanization gradient. *K*‐means clustering (*K* = 3) was performed using site scores on these two PCA axes to classify the 30 sampling sites into three urbanization levels—urban, suburban, and rural—allowing us to assess differences in community diversity across gradient levels. PCA scores used for clustering analysis are provided in Data S2. Boxplots were used to visualize the distribution of diversity indices across the three urbanization classes, with mean, median, and sample sizes indicated.

To further quantify the effects of urbanization on animal community diversity, generalized linear models (GLMs) were constructed. To avoid potential bias caused by differences in species richness between birds and mammals, the models were built separately for the overall community, ground‐dwelling birds, and mammals, with Shannon, FDis, and PD as response variables. Gaussian error distribution and identity link functions were specified, which are statistically equivalent to linear regression. Predictor variables were selected based on ecological relevance and correlation screening. Candidate models were selected using stepwise AICc, and models with ΔAICc < 2 were retained for model averaging (Burnham and Anderson 2004). Model averaging and coefficient estimation were conducted using the MuMIn R package, with significance set at *p* < 0.05. The dataset used for GLM analyses, including all response and predictor variables, is available in Data S3.

To examine the relationships between species functional traits and environmental gradients, we conducted RLQ analysis (Dolédec et al. 1996), integrating three matrices: environmental variables (*R*), species abundance (*L*), and species traits (*Q*). The *R* matrix included urbanization pressure variables (DGS, BD, BH, RD, and POD) and local habitat structure variables (WC, CC, GC, and NT). Urbanization variables were selected based on their relevance to urban intensity and their explanatory performance in preliminary analyses, while local habitat variables were included to represent site‐level habitat structure; the *L* matrix represented species abundance of ground‐dwelling birds and mammals; and the *Q* matrix contained four traits. Following the standard RLQ procedure, PCA was applied to the *R* matrix, correspondence analysis (CA) to the *L* matrix, and Hill–Smith analysis to the *Q* matrix prior to RLQ integration (Dolédec et al. 1996). Here, the PCA on the *R* matrix served as an internal ordination step within the RLQ framework and was independent of the earlier PCA used to derive the urbanization gradient for site classification. The RLQ analysis incorporated both macro‐scale urbanization pressures and local habitat characteristics to enhance ecological interpretation. Monte Carlo permutation tests (*n* = 99,999) were used to assess the significance of trait–environment relationships under Model 2 (effects of environment on traits) and Model 4 (trait effects on community structure). The *R*, *L*, and *Q* matrices used in the RLQ analysis are provided in Data S4.

**FIGURE 2:**
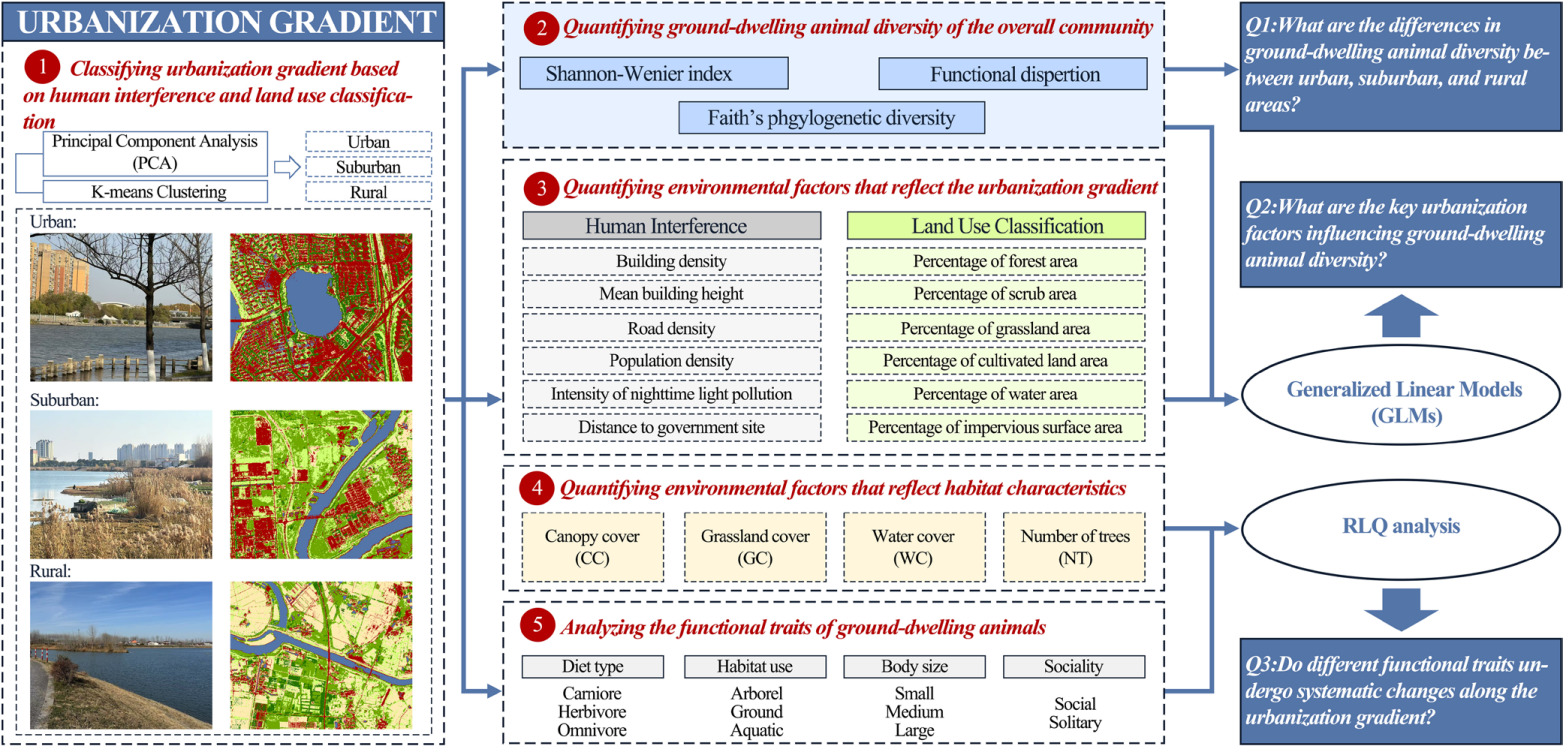
Research framework illustrating the study design and analytical approach.

To control for multicollinearity, we calculated variance inflation factors (VIF) using the usdm package, retaining only those variables with VIF < 10 and ecological interpretability for modeling (Zuur et al. 2010). All statistical analyses were performed using R software version 4.3.3 (R Core Team 2023) (Figure 2). All analyses were conducted in R, and the full script is provided in Methods S1.

## 3 Results

### 3.1 Species Composition of Ground‐Dwelling Animals

**FIGURE 3:**
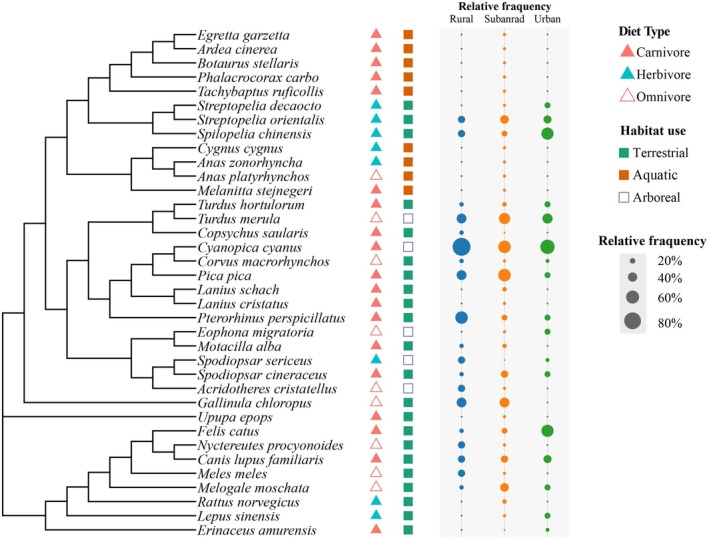
Phylogeny and guild composition of the ground‐dwelling animals. Their relative frequency was also noted in the figure.

A total of 36 ground‐dwelling bird and mammal species were recorded, encompassing 10 orders, 21 families, and 27 genera. Birds dominated the assemblage, with 28 species (77.8%) from 15 families and 22 genera, whereas mammals comprised 8 species (22.2%) from 6 families and 8 genera. Dietary guilds included 8 herbivores, 19 carnivores, and 9 omnivores, suggesting notable trophic diversity along the Chuhe River corridor. Species also exhibited varied habitat‐use strategies—arboreal, aquatic, and terrestrial—indicating high ecological heterogeneity across sites (Figure 3).

The most frequently detected species was the Azure‐winged Magpie ( *Cyanopica cyanus* ), recorded at 20 sampling sites (66.7%). This was followed by the Chinese Blackbird ( *Turdus merula* , 14 sites, 46.7%), Eurasian Magpie ( *Pica pica* , 13 sites, 43.3%), Oriental Turtle Dove ( *Streptopelia orientalis* , 10 sites, 33.3%), Spotted Dove ( *Spilopelia chinensis* , 10 sites, 33.3%), Masked Laughingthrush (*Pterorhinus perspicillatus*, 9 sites, 30.0%), Common Moorhen ( *Gallinula chloropus* , 9 sites, 30.0%), as well as cats ( *Felis catus* ) and dogs ( *Canis lupus familiaris* ), both recorded at 9 sites (30.0%). In addition, small mammal species such as the Chinese ferret‐badger ( *Melogale moschata* ) and the Chinese Hare ( *Lepus sinensis* ) were recorded at multiple sampling sites, indicating that mammals still maintain a certain level of activity within urban green spaces. Some less frequently observed but ecologically significant species included wetland‐dependent ground‐dwelling birds such as the Little Egret ( *Egretta garzetta* ), Common Cormorant ( *Phalacrocorax carbo* ), and Grey Heron ( *Ardea cinerea* ).

Notably, species that are relatively uncommon in urbanized Nanjing—such as the Eurasian bittern ( *Botaurus stellaris* ) and Eurasian hoopoe ( *Upupa epops* )—were also detected in this study. Their presence suggests that the Chuhe River riparian zone may serve important habitat or stopover functions for migratory or cryptic ground‐dwelling bird species.

### 3.2 Diversity Patterns Along the Urbanization Gradient

**FIGURE 4:**
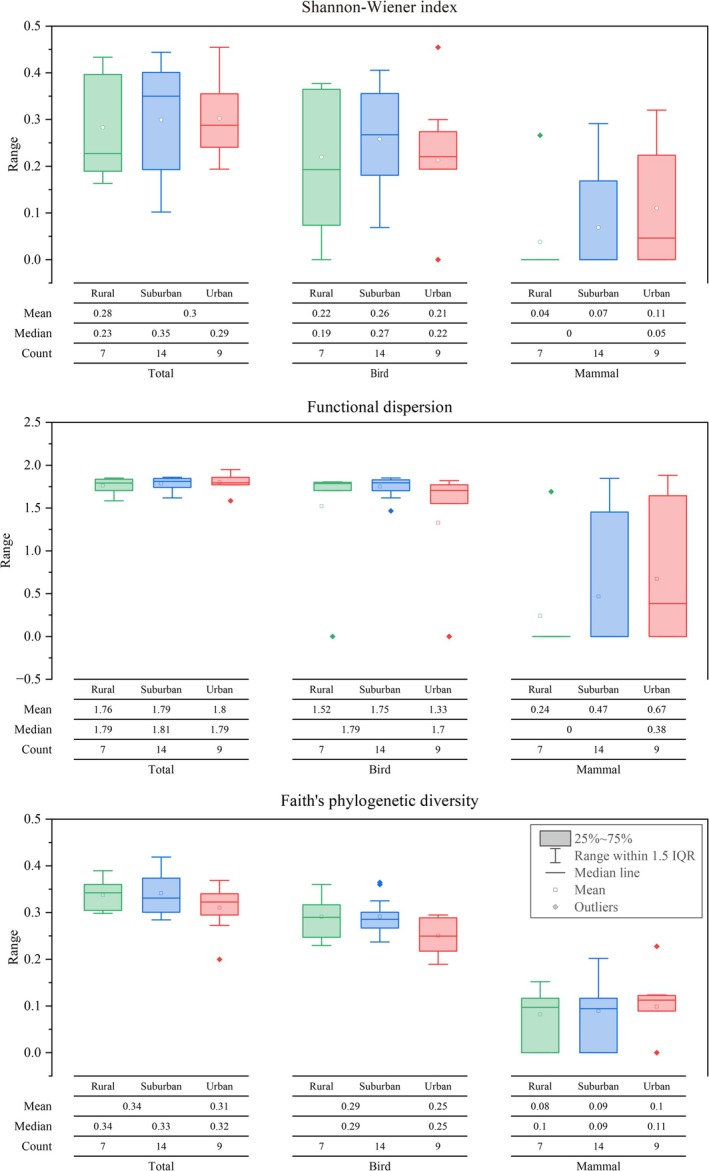
Distribution of Shannon–Wiener diversity, functional dispersion, and Faith's phylogenetic diversity of ground‐dwelling animal communities across urban, suburban, and rural sites. No significant differences were detected among urbanization levels (Kruskal–Wallis tests, *p* > 0.05).

As shown in Figure 4, Shannon diversity varied among rural, suburban, and urban areas for the overall ground‐dwelling animal community, birds, and mammals. For total communities, median Shannon values were slightly higher in suburban sites than in rural and urban sites. Bird communities also showed higher median Shannon diversity in suburban areas, whereas mammal communities generally exhibited lower Shannon values with relatively higher medians in urban sites. However, Kruskal–Wallis tests revealed no significant differences in Shannon diversity among urbanization levels for total communities, birds, or mammals (all *p* > 0.05).

FDis of the overall ground‐dwelling animal community, birds, and mammals showed only minor variation among urbanization levels (Figure 4), and no significant differences were detected for any group (all *p* > 0.05).

PD of total communities, birds, and mammals similarly exhibited limited variation across rural, suburban, and urban categories. Although median PD values differed slightly among groups, Kruskal–Wallis tests indicated that these differences were not statistically significant (all *p* > 0.05).

### 3.3 Effects of Urbanization Factors on Ground‐Dwelling Animal Diversity

GLMs results showed distinct effects of urbanization variables on different dimensions of animal community diversity. Across the nine diversity indices, several urbanization factors were retained in the best models (ΔAICc < 2), with varying directions and significance levels (Table 1).

**TABLE 1:** Model‐averaged effects of urbanization‐related variables on diversity indices of ground‐dwelling bird and mammal communities.

Variable	Total			Bird			Mammal		
	Shannon	FDis	PD	Shannon	FDis	PD	Shannon	FDis	PD
NLP	/	/	/	/	/	/	−0.09 (−0.06)	/	/
POD	/	/	/	/	−0.39 (0.23).	/	/	−0.83 (0.36)*	/
RD	/	/	/	/	/	−0.04 (0.02)*	0.21 (0.06)***	0.58 (0.32).	0.05 (0.03)*
DGS	−0.10 (−0.04)*	−0.08 (0.03)*	−0.03 (−0.02)	−0.14 (0.06)*	/	−0.05 (0.02)**	/	/	/
BD	/	−0.08 (0.03)*	−0.05 (0.020)*	/	/	−0.03 (0.02).	/	/	/
BH	/	0.10 (0.04)**	/	−0.12 (0.056)*	−0.46 (0.24).	/	/	/	/
IAP	/	/	/	/	/	/	/	0.96 (0.39)*	/
CAP	/	/	/	/	−0.52 (0.21)*	/	/	/	/
SAP	/	/	/	/	/	0.04 (0.02)*	/	/	/
FAP	/	/	/	/	/	/	/	/	/
*R* ^2^	0.17	0.39	0.20	0.21	0.33	0.47	0.37	0.35	0.13

*Note:* Model‐averaged standardized regression coefficients (±SE) from GLMs examining the effects of urbanization‐related variables on Shannon diversity, functional dispersion (FDis), and phylogenetic diversity (PD) of total, bird, and mammal communities. Model averaging was conducted across candidate models with ΔAICc < 2. Values in parentheses indicate standard errors, and “/” denotes variables not retained in the averaged models. Significance levels are indicated as “***” *p* < 0.001, “**” *p* < 0.01, “*” *p* < 0.05, and “·” *p* < 0.1. Values without symbols indicate non‐significant effects (*p* > 0.05). *R* ^2^ represents the explained variance of each model.

Abbreviations: Urbanization‐related variables—BD = building density, BH = mean building height; percentage of impervious surface area, CAP = percentage of cultivated land area, DGS = distance to the government center, FAP = percentage of forest area, NLP = intensity of nighttime light pollution, POD = population density, RD = road density, SAP = percentage of scrub area.

Model fit (*R* ^2^), derived from the Gaussian GLMs, varied among indices, being highest for bird phylogenetic diversity (*R* ^2^ = 0.47) and lowest for mammals (*R* ^2^ = 0.12), suggesting differing sensitivities of animal groups to urbanization drivers.

**FIGURE 5:**
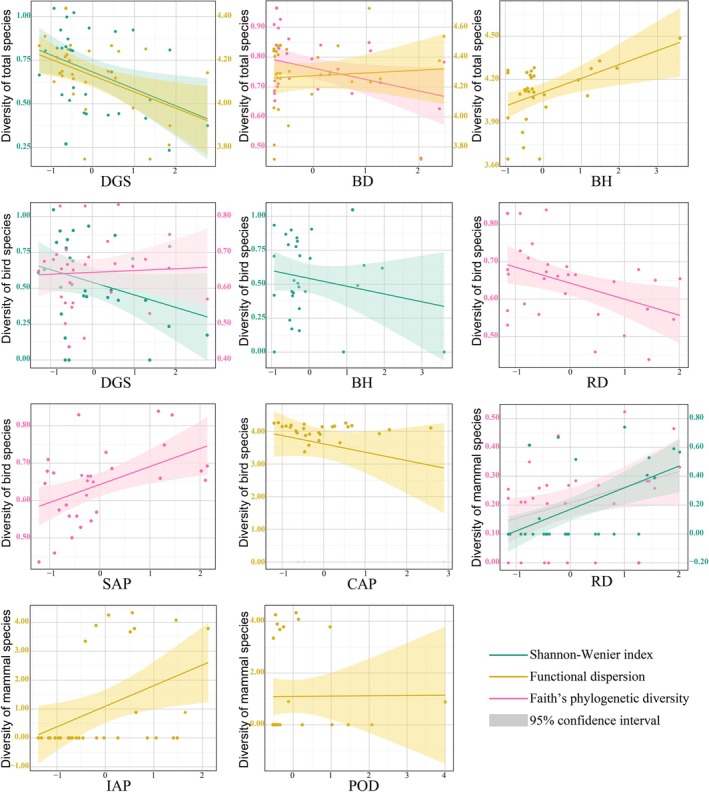
Using GLMs to analyze the relationships between urbanization factors and animal diversity indices. Subplot grouping: Upper panels = diversity of total species (bird + mammal); middle panels = bird diversity; lower panels = mammal diversity. Axes: *x*‐axis = environmental factors (BD = building density, BH = mean building height, DGS = distance to government site, CAP = percentage of cultivated land area, IAP = percentage of impervious surface area, POD = population density, RD = road density, SAP = percentage of scrub area); *y*‐axis = diversity of the corresponding taxon.

As illustrated in Figure 5, for the overall ground‐dwelling animal community, DGS showed a significant negative effect on both species Shannon diversity and FDis, indicating their gradual decline from the urban core. BD showed a significant negative effect on FDis and PD, whereas BH had a significant positive effect on FDis.

Within the ground‐dwelling bird community, the negative effect of DGS was also significant, impacting both Shannon and PD. FDis was jointly influenced by multiple urbanization variables, including POD, BH, and CAP. Bird phylogenetic diversity was positively associated with SAP and negatively associated with RD.

For mammal communities, RD exhibited positive effects on several diversity indices, possibly reflecting that urban road edges provide new habitats or movement corridors for certain small‐ to medium‐sized mammals. However, POD had a significant negative effect on FDis, while IAP was positively related to FDis. NLP showed a marginally significant negative impact on mammal Shannon diversity.

### 3.4 Functional Trait Responses to the Urbanization Gradient

**FIGURE 6:**
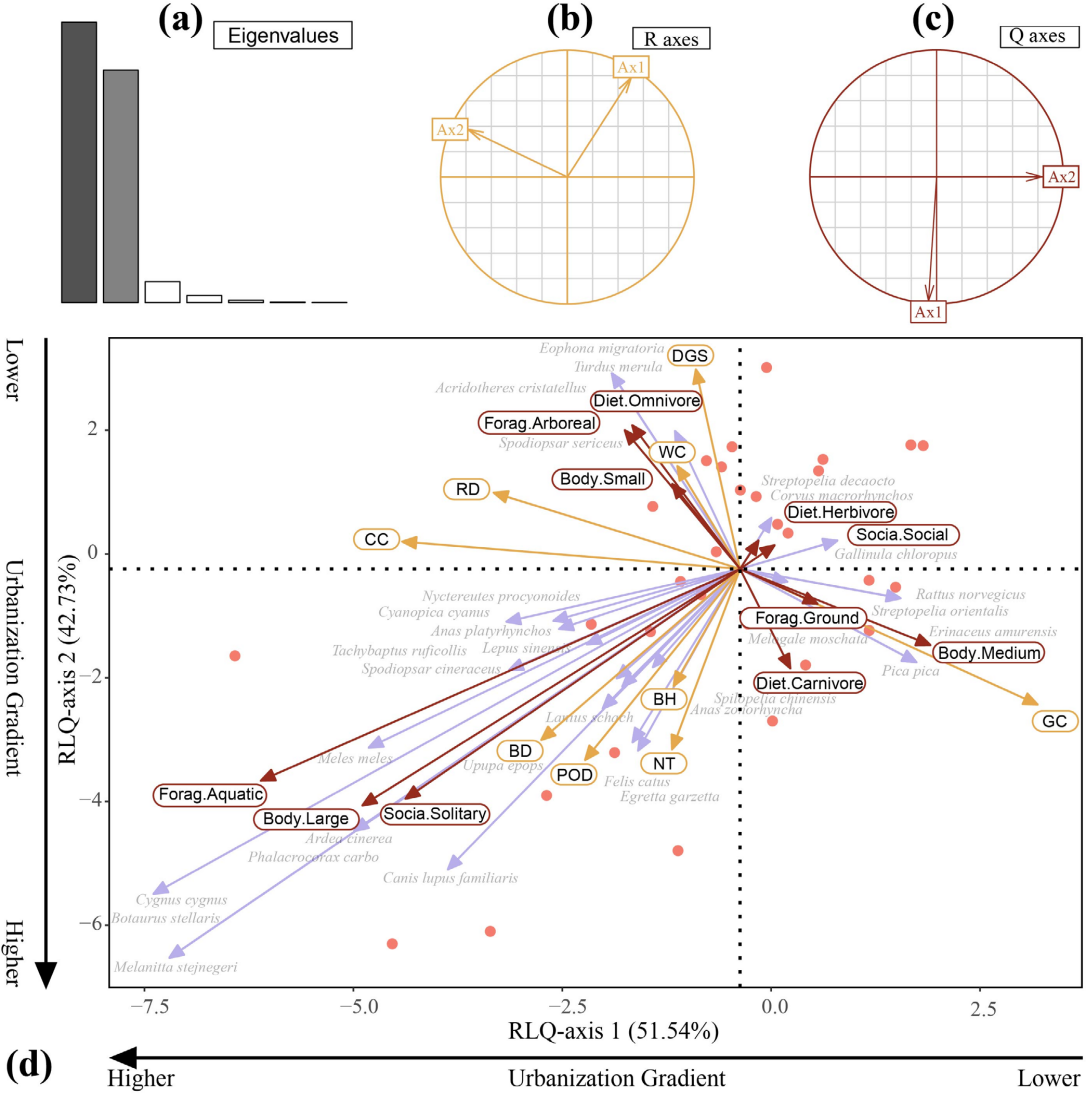
RLQ analysis reveals the relationship between ground‐dwelling animal functional traits and urbanization factors. (a) Eigenvalues Plot (b) Projection of Environmental Axes (*R* axes) (c) Projection of Trait Axes (*Q* axes) (d) RLQ Ordination Plot (Species × Traits × Environment): Yellow arrows represent environmental variables (*R*), purple arrows represent species scores (*L*), and red arrows represent functional traits (*Q*). Sampling sites are indicated by different symbols: red circles represent urban sites, blue squares represent suburban sites, and green triangles represent rural sites. BD = building density, BH = mean building height, CC = canopy cover, DGS = distance to government site, GC = grassland cover, NT = the number of trees, POD = population density, RD = road density, WC = water cover.

As shown in Figure 6, the RLQ analysis revealed significant structured associations between the functional traits of ground‐dwelling animals and environmental variables. The first two RLQ axes explained 51.54% and 42.73% of the total inertia, respectively, representing the primary gradients linking traits and environmental factors. The significance test of the RLQ model indicated that model 4 was significant (*p* = 0.02), suggesting that the distribution of functional traits in ground‐dwelling animal communities along the urbanization gradient is non‐random and associated with environmental variation. However, model 2 was not significant, indicating that the relationship between traits and species composition alone was not strong. Within the RLQ framework, model 4 tests the overall coupling between environmental gradients and trait distributions mediated by species composition, whereas model 2 evaluates only the trait–species matrix independently. Therefore, the significant result of model 4, despite the non‐significant model 2, suggests that environmental filtering may operate indirectly through species turnover along the urbanization gradient.

In the RLQ biplot space, sampling sites exhibited a clear pattern corresponding to the urbanization gradient. Urban sites were mainly clustered in the negative quadrant of both axes, corresponding to highly disturbed and structurally complex environments. Rural sites were located in the positive quadrant, reflecting low‐disturbance, more natural ecological conditions. Suburban sites were positioned in intermediate areas or near the intersection of the two axes, indicating transitional habitat characteristics. This spatial pattern corresponded well with the urban–suburban–rural classification obtained from k‐means clustering, highlighting the correspondence between urbanization level and functional trait structure.

Environmental variables also showed clear differentiation in the RLQ space. Variables such as CC, WC, RD, and DGS were primarily associated with the natural side of the gradient, whereas BD, BH, and POD clustered on the urban end. GC was positioned in the transitional zone, reflecting the characteristics of moderately urbanized environments.

Functional traits of animals exhibited distinct responses along the RLQ axes. Social and herbivorous species were predominantly associated with more natural sites, while omnivorous, small‐bodied, and arboreal species were more common in suburban areas. Urban sites were mainly associated with aquatic‐foraging, large‐bodied, and solitary species. Some carnivorous and ground‐foraging species were distributed across both urban–rural edges and rural areas.

## 4 Discussion

### 4.1 Responses of Ground‐Dwelling Animal Diversity to the Urban–Suburban–Rural Gradient

Using the categorical urban–suburban–rural gradient, we examined variations in α diversity across ground‐dwelling animal communities. No statistically significant differences were detected in Shannon diversity, functional diversity (FDis), or phylogenetic diversity (PD) among urbanization levels for total communities, birds, or mammals (all Kruskal–Wallis tests, *p* > 0.05; Figure 4).

Although median values of Shannon diversity for total communities and birds were slightly higher in suburban sites, and mammal Shannon diversity tended to be higher in urban areas, these variations were not statistically significant. Similarly, FDis and PD showed only minor variation across rural, suburban, and urban sites for all taxonomic groups, and none of these differences reached statistical significance.

These results indicate that, at the scale of broad urbanization categories, local α diversity of ground‐dwelling animals remained relatively stable along the urban–suburban–rural gradient. Similar patterns have been reported in urban ecological studies, where local diversity metrics exhibit weak or inconsistent responses to coarse urbanization gradients despite underlying changes in community composition and functional structure (McKinney 2008; Aronson et al. 2014).

Consequently, the absence of significant differences in α diversity does not imply a lack of urbanization effects, but rather suggests that the influence of urbanization may not be adequately captured by categorical gradients alone, highlighting the need to examine specific urbanization‐related variables operating along continuous gradients, as addressed in the following section.

### 4.2 Key Urbanization Drivers of Diversity Changes

This study evaluated the effects of multiple urbanization‐related variables on species, functional, and phylogenetic diversity of ground‐dwelling bird and mammal communities.

To further identify the underlying drivers of diversity variation, GLMs were used to quantify the relationships between specific urbanization‐related variables and different dimensions of diversity. The GLM results revealed that, despite the absence of significant differences in α‐diversity across broad urbanization categories (Section 4.1), several specific urbanization‐related factors exerted significant and taxon‐specific effects on diversity metrics. These results highlight the complexity of urban environment–community interactions and suggest that urbanization impacts may be more effectively captured by continuous variables rather than categorical gradients.

Overall, ground‐dwelling bird diversity showed higher sensitivity to urban‐related pressures, whereas mammal communities exhibited certain ecological adaptability and characteristics commonly associated with “urban winners.” Different urbanization‐related variables influenced species diversity, functional diversity, and phylogenetic diversity in distinct ways across taxa, underscoring the heterogeneous nature of biodiversity responses to urbanization.

Population density generally exerted negative effects on functional diversity, significantly reducing functional heterogeneity in both bird and mammal communities. This likely indicates that high human activity intensity compresses species ecological niche space, limiting the diversity of ecological strategies (Zhang et al. 2020), and also reflects the phenomenon of species homogenization in densely populated urban areas.

Road density exhibited contrasting effects between the two taxa. Phylogenetic diversity of ground‐dwelling birds was significantly negatively correlated with road density, suggesting that infrastructure may constrain the habitat of more distantly related species, leading to phylogenetic clustering (Wang et al. 2024). In contrast, mammal communities showed positive correlations between road density and Shannon diversity, functional diversity, and phylogenetic diversity, implying that some urban‐adapted species (e.g., cats, rodents) may utilize roadside habitats and even disperse along roads (Gaynor et al. 2020).

Building height had negative effects on both Shannon and functional diversity of ground‐dwelling birds, possibly related to the increased human disturbance associated with high‐rise buildings, such as noise, traffic, and human presence (Leveau and Leveau 2016). Additionally, the risk of bird collisions with glass facades in tall buildings may exacerbate survival pressures for ground‐dwelling birds in urban cores (Van Doren et al. 2021; Loss et al. 2014).

Distance to government site, used as a proxy for spatial urbanization gradient, negatively influenced Shannon diversity of overall and ground‐dwelling bird communities. This suggests that although urban edges possess some natural attributes, edge effects and habitat fragmentation may similarly simplify community structure (Hastedt and Tietze 2023; Leveau and Leveau 2016).

Regarding land cover, the percentage of scrub area had a positive effect on bird phylogenetic diversity, indicating the important buffering role of natural vegetation in maintaining phylogenetic diversity (Lopes et al. 2023). Conversely, the percentage of cultivated land area was significantly negatively correlated with bird functional diversity, suggesting higher functional redundancy and ecological simplification of bird communities in agricultural landscapes (Etard et al. 2022).

Percentage of impervious surface area had no significant impact on birds but showed a positive effect on mammal functional diversity. This may reflect the high ecological plasticity of urban‐adapted mammals (such as omnivorous small‐ and medium‐sized species) that maintain diverse functional strategies even within highly artificial urban cores (McKinney 2008; Bateman and Fleming 2012).

### 4.3 Functional Trait Filtering Effects During Urbanization

The RLQ analysis provided partial support for the influence of urbanization on the functional trait composition of ground‐dwelling animal communities, suggesting a non‐random structuring of traits along the urbanization gradient. In the RLQ framework, Model 2 evaluates the association between species composition and functional traits, whereas Model 4 tests the overall coupling between environmental variables and trait distributions mediated by species composition. In this study, Model 2 was not significant, indicating a weak direct structure between traits and species composition alone. However, Model 4 was significant, suggesting that functional traits are indirectly structured along environmental gradients through species turnover. This pattern implies that environmental gradients associated with urbanization may exert an indirect filtering effect on trait composition.

Functional traits exhibited non‐random spatial distribution patterns along the urbanization gradient, reflecting the combined influence of habitat heterogeneity and disturbance intensity on community assembly. Similar patterns have been reported in previous studies of urban animal communities (Sol et al. 2013; Aronson et al. 2014), providing broader empirical support for trait‐based responses across urban–rural systems.

Specifically, urban areas were characterized by a higher prevalence of solitary behavior, large body size, and aquatic activity traits, suggesting that species persisting in highly artificial environments may be shaped more by ecological constraints and behavioral flexibility rather than rapid morphological evolution (Santini et al. 2019). Notably, although small body size has often been described as an urban‐adapted trait in previous studies (Neate‐Clegg et al. 2023), our results indicate that body size responses are context‐dependent across the urban–suburban–rural gradient. In this system, smaller‐bodied species were more frequent in suburban environments, whereas urban cores were dominated by larger‐bodied taxa, potentially reflecting differences in habitat structure, resource distribution, and disturbance regimes across gradient positions. In contrast, social and herbivorous species were primarily associated with rural and low‐disturbance sites, suggesting higher sensitivity to habitat simplification and stronger dependence on intact ecological structures. Suburban sites exhibited higher functional trait diversity and more heterogeneous trait composition, indicating that intermediate disturbance levels may promote the coexistence of multiple ecological strategies and act as important transition zones in urban landscapes.

### 4.4 Study Limitations

Despite the insights provided by this study, several limitations should be acknowledged.

First, the dataset contains a relatively high proportion of singleton and site‐unique species, which may affect the robustness of certain diversity metrics, particularly Shannon diversity and functional dispersion. Rare species can exert a disproportionate influence on diversity estimates, potentially amplifying stochastic variation in community‐level indices. Therefore, the observed patterns should be interpreted with appropriate caution. In addition, mammal species richness in this study was relatively low, and urban mammal assemblages were dominated by a small number of synanthropic species, which may influence Shannon diversity and functional trait metrics and warrant cautious interpretation of mammal‐specific patterns. Nevertheless, such rarity patterns are commonly reported in urban ecological surveys and reflect the inherently heterogeneous and fragmented nature of urban habitats, especially along urban–rural transition zones.

Second, this study was conducted during a single season (winter) and within a specific landscape context, namely a riparian corridor. Winter is not the optimal period for capturing peak vegetation structure and productivity, and seasonal dormancy may lead to an underestimation of vegetation cover and complexity, particularly for herbaceous components. However, conducting vegetation surveys during winter also reduces seasonal noise associated with short‐term phenological dynamics and transient vegetation growth. As a result, the measured habitat variables primarily reflect persistent structural features, such as canopy cover and tree abundance, rather than short‐term fluctuations in vegetation biomass. These stable structural attributes are especially relevant to ground‐dwelling animal activity during winter and provide a consistent basis for linking habitat structure with species occurrence and functional traits.

Nevertheless, seasonal variation in species activity, detectability, and resource availability, as well as the ecological distinctiveness of river‐adjacent urban systems, may limit the generality of our findings. Accordingly, the conclusions drawn here are most applicable to winter conditions along urban–suburban–rural gradients within riparian landscapes and should not be extrapolated to other seasons or landscape types without caution. Future studies incorporating multi‐season vegetation surveys and year‐round wildlife monitoring across diverse urban landscape contexts would help to further test and generalize the patterns observed in this study.

## 5 Conclusion

This study comprehensively evaluated the responses of ground‐dwelling animal community structure and functional traits along an urbanization gradient using multidimensional diversity indices, including taxonomic, functional, and phylogenetic diversity. The main findings are as follows:

1. No significant differences in α diversity were detected among rural, suburban, and urban sites for total ground‐dwelling animal communities, birds, or mammals. Shannon diversity, functional diversity, and phylogenetic diversity all exhibited relatively stable patterns across the categorical urban–suburban–rural gradient, indicating that coarse urbanization classifications alone may be insufficient to capture diversity responses.
2. In contrast, analyses based on specific urbanization‐related variables revealed significant and taxon‐specific effects on multiple dimensions of diversity. Variables such as population density and building height significantly compressed niche breadth and phylogenetic breadth of ground‐dwelling birds, whereas road density and percentage of impervious surface area may provide alternative habitats for mammals.
3. RLQ analysis indicated a significant overall coupling between urbanization‐related environmental gradients and animal functional traits (Model 4), suggesting that trait distributions are indirectly structured along urbanization gradients through species turnover. However, the lack of significance in Model 2 implies that traits are not strongly structured by species composition alone. Together, these results suggest that environmental filtering may operate indirectly in urban ecosystems, rather than producing a direct trait–species structure. Functional traits such as body size, sociality, and foraging strategy showed clear spatial turnover along the urban gradient, reflecting consistent but context‐dependent ecological responses to urbanization.

Based on these findings, urban ecological management and spatial planning should prioritize the conservation and restoration of structurally complex and habitat‐heterogeneous patches, enhance green network connectivity, and maintain ecological permeability across urban landscapes. Particular attention should be given to suburban areas due to their strategic position in urban ecological spatial structure, and they should be treated as priority zones for careful management during urban expansion. Additionally, conservation strategies should explicitly account for the contrasting responses of ground‐dwelling birds and mammals in order to reduce risks of functional homogenization and phylogenetic simplification during urbanization.

As urbanization in China transitions from rapid expansion to a more stabilized phase, continued pressure from urban edge expansion remains a key driver of biodiversity change. Future urban development should emphasize ecological security patterns, regulate expansion intensity, optimize land‐use configuration, and promote urban renewal guided by ecological resilience and green infrastructure planning.

## Author Contributions

**Wenjing Chen:** conceptualization (equal), data curation (equal), formal analysis (equal), investigation (equal), supervision (equal), validation (equal), writing – original draft (equal). **Qi Zhu:** investigation (equal), supervision (equal), validation (equal), visualization (equal), writing – original draft (equal), writing – review and editing (equal). **Yunfeng Yang:** conceptualization (equal), funding acquisition (equal), methodology (equal), project administration (equal), resources (equal).

## Funding

This work was supported by the National Natural Science Foundation of China (grant number: 32171859).

## Conflicts of Interest

The authors declare no conflicts of interest.

## Supporting information


**Data S1:** Phylogenetic tree in Newick format.
**Data S2:** PCA scores used for clustering analysis.
**Data S3:** Species abundance and diversity dataset used for GLM analyses. *R*, *L*, and *Q* matrices used for RLQ analysis.
**Data S4:** *R*, *L*, and *Q* matrices used for RLQ analysis.
**Methods S1**. R script (S1.R) containing all analyses performed in this study.

## Data Availability

All the required data are uploaded as Supporting Information.
